# Supplementary material for: Induction of Metabolic Changes in Amino Acid, Fatty Acid, Tocopherol, and Phytosterol Profiles by Exogenous Methyl Jasmonate Application in Tomato Fruits
Source: Plants (Basel). 2022 Jan 28;11(3):366. doi: 10.3390/plants11030366 (PMC8838126; doi:10.3390/plants11030366)
Supplement: Supplementary file 1 [file plants-11-00366-s001.zip › plants-1525363-SI/Table S1_reviewed.pdf]

Table S1. Primary metabolites in tomato (*Solanum lycopersicum* L. cv. Grape) treated with ethylene (ETHY) and methyl jasmonate (MeJA) compared to control group (CTRL) detected by gas chromatography-mass spetrometry (GC-MS).

**Metabolite**	**04 DAH**			**10 DAH**			**21 DAH**		
	CTRL	***ETHY***	***MeJA***	***CTRL***	***ETHY***	***MeJA***	***CTRL***	***ETHY***	***MeJA***
**A) Sugars**									
Glucose	1534.5 ± 76.0 ^{f}	4101.0 ± 228.0 ^{d}	2225.7 ± 107.5 ^{e}	1977.6 ± 11.4 ^{ef}	9991.1 ± 130.2 ^{b}	4489.9 ± 24.7 ^{d}	4352.0 ± 281.0 ^{d}	11543.0 ± 359.0 ^{a}	8335.5 ± 176.6 ^{c}
Fructose	27474.0 ± 4039.0 ^{c}	95020.0 ± 3675.0 ^{g}	45970.0 ± 3735.0 ^{f}	59266.0 ± 16310.0 ^{e}	115707.0 ± 714.0 ^{b}	73375.0 ± 1137.0^{d}	101194.0 ± 5662.0 ^{c}	136003.0 ± 5835.0 ^{a}	95526.0 ± 11592.0 ^{c}
Sucrose	38205.0 ± 596.0 ^{g}	69829.0 ± 4129.0 ^{d}	49135.0 ± 2493.0 ^{f}	54654.0 ± 716.0 ^{ef}	93085.0 ± 881.0 ^{b}	60830.0 ± 367.0 ^{e}	84839.0 ± 4545.0 ^{c}	123867.0 ± 4068.0 ^{a}	81355.0 ± 2080.0 ^{c}
Allose	1098.6 ± 42.2 ^{e}	1536.5 ± 33.8 ^{d}	1164.2 ± 44.6 ^{e}	1563.4 ± 21.5 ^{d}	4212.4 ± 103.8 ^{b}	1397.8 ± 35.4 ^{de}	3309.0 ± 380.0 ^{c}	6571.0 ± 202.0 ^{a}	4243.5 ± 106.5 ^{b}
Gulose	221.5 ± 9.78 ^{g}	397.2 ± 11.9 ^{g}	244.6 ± 10.7 ^{h}	790.4 ± 38.3 ^{e}	1095.4 ± 7.74 ^{b}	712.9 ± 25.5 ^{f}	1017.0 ± 60.4 ^{c}	1339.4 ± 42.8^{a}	911.8 ± 23.5 ^{d}
Glucaric acid	42.2 ± 1.61 ^{e}	81.1 ± 2.24 ^{cd}	46.5 ± 3.67 ^{e}	71.9 ± 1.30 ^{d}	128.7 ± 1.63 ^{b}	89.9 ± 2.39 ^{c}	124.6 ± 8.70 ^{b}	206.4 ± 6.98 ^{a}	133.0 ± 3.25 ^{b}
Myo-inositol	77.9 ± 2.91 ^{ef}	112.6 ± 1.72 ^{e}	73.4 ± 2.82 ^{f}	169.6 ± 2.04 ^{d}	229.3 ± 9.72 ^{c}	165.8 ± 3.44 ^{d}	340.1 ± 3.44^{b}	673.8 ± 11.4 ^{a}	367.4 ± 39.7 ^{b}
Mannose	42.2 ± 3.10 ^{f}	86.2 ± 5.01 ^{d}	58.6 ± 4.34 ^{e}	100.0 ± 2.30 ^{c}	139.4 ± 2.03 ^{b}	97.9 ± 1.51 ^{c}	142.4 ± 11.0 ^{b}	249.6 ± 2.33 ^{a}	146.6 ± 4.90 ^{b}
Ribose	174.6 ± 7.42 ^{f}	217.4 ± 6.14 ^{e}	165.9 ± 6.8 ^{f}	249.4 ± 3.91 ^{d}	307.8 ± 10.00 ^{c}	221.2 ± 2.16 ^{e}	386.4 ± 27.0 ^{b}	530.8 ± 11.7 ^{a}	372.5 ± 7.75 ^{b}
Arabino-furanose	15.1 ± 0.74 ^{f}	21.7 ± 0.62 ^{e}	14.0 ± 0.45 ^{f}	25.5 ± 0.84 ^{e}	68.2 ± 3.77 ^{b}	27.3 ± 0.49 ^{e}	45.2 ± 3.28 ^{d}	104.1 ± 2.88 ^{a}	60.8 ± 5.20 ^{c}
Total	68885.0 ± 4082.0 ^{h}	171403.0 ± 6682.0 ^{d}	99098.0 ± 5695.0 ^{g}	118868.0 ± 5993.0 ^{f}	224964.0 ± 1220.0 ^{b}	141408.0 ± 1197.0 ^{e}	195750.0 ± 9973.0 ^{c}	281088.0 ± 10338.0 ^{a}	191152.0 ± 2920.0 ^{c}
**B) Organic acids**									
Oxaloacetic acid	573.3 ± 24.3 ^{g}	1321.4 ± 32.5 ^{de}	1352.7 ± 64.4 ^{d}	2380.4 ± 56.5 ^{c}	6070.9 ± 28.1 ^{b}	9559.7 ± 56.9 ^{a}	1241.9 ± 80.7$^{d e}$	1203.3 ± 64.1 ^{e}	911.5 ± 25.1 ^{f}
Citric acid	6517.0 ± 413.0 ^{g}	11256.0 ± 290.0 ^{ef}	10808.0 ± 505.0 ^{ef}	7878.0 ± 457.0 ^{f}	38455.0 ± 3291.0 ^{b}	74269.0 ± 6427.0 ^{a}	18901.0 ± 1208.0 ^{d}	16520.0 ± 1124.0 ^{de}	25977.0 ± 655.0 ^{c}
Succinic acid	2646.0 ± 360.0 ^{e}	6886.0 ± 519.0 ^{d}	8590.0 ± 386.0 ^{d}	12894.0 ± 485.0 ^{c}	26896.0 ± 1390.0 ^{b}	55839.0 ± 2291.0 ^{a}	2862.0 ± 254.0 ^{e}	4309.2 ± 183.6 ^{e}	4391.9 ± 106.3 ^{e}
Aconitic acid	61.1 ± 2.90 ^{e}	100.3 ± 4.02 ^{c}	64.9 ± 2.47 ^{e}	83.1 ± 0.86 ^{g}	111.7 ± 5.75 ^{b}	74.7 ± 4.14 ^{d}	101.4 ± 5.70 ^{c}	137.9 ± 5.37 ^{a}	100.0 ± 1.73 ^{c}
Malic acid	2537.5 ± 101.3 ^{g}	9927.0 ± 280.0 ^{e}	13126.0 ± 502.0 ^{d}	6653.7 ± 174.0 ^{f}	22582.0 ± 316.0 ^{b}	53604.3 ± 103.0 ^{a}	16800.0 ± 1014.0 ^{c}	16511.0 ± 460.0 ^{c}	17357.0 ± 308.0 ^{c}
Citraconic acid	17.4 ± 0.74 ^{d}	19.8 ± 2.00 ^{d}	71.1 ± 6.39 ^{c}	104.5 ± 12.4 ^{a}	85.3 ± 3.45 ^{b}	79.3 ± 5.44 ^{bc}	101.9 ± 6.07 ^{a}	105.4 ± 3.44 ^{a}	110.3 ± 1.75 ^{a}
Fumaric acid	167.9 ± 6.66 ^{e}	170.8 ± 3.76 ^{e}	178.4 ± 2.07 ^{e}	181.8 ± 1.17 ^{de}	217.9 ± 9.13 ^{b}	234.7 ± 4.53 ^{a}	237.8 ± 10.4 ^{a}	196.9 ± 8.40 ^{cd}	201. ± 4.80 ^{c}
Propanoic acid	111.7 ± 6.77 ^{c}	114.5 ± 5.59 ^{c}	99.5 ± 6.91 ^{c}	145.6 ± 1.50 ^{c}	138.3 ± 0.86 ^{c}	132.9 ± 0.51 ^{c}	458.6 ± 79.1 ^{b}	412.5 ± 37.0 ^{b}	2062.1 ± 193.0 ^{a}
Butanoic acid	284.5 ± 18.9 ^{fg}	268.5 ± 18.6 ^{g}	460.6 ± 34.1 ^{de}	393.4 ± 10.9 ^{ef}	369.7 ± 2.77 ^{efg}	540.7 ± 4.82^{cd}	641.8 ± 96.9 ^{bc}	690.9 ± 56.8 ^{b}	1879.7 ± 92.2 ^{a}
Total	12917.0 ± 804.0 ^{f}	30065.0 ± 567.0 ^{e}	34752.0 ± 1341.0 ^{de}	30715.0 ± 907.0 ^{e}	94927.0 ± 3789.0 ^{b}	194334.0 ± 8679.0 ^{a}	41346.0 ± 2535.0 ^{d}	40087.0 ± 1720.0 ^{d}	52991.0 ± 1021.0 ^{c}
**C) Amino acids**									
Proline	501.2 ± 14.8 ^{f}	974.5 ± 62.4 ^{ef}	5207.0 ± 259.0^{b}	1167.2 ± 26.8^{e}	2266.1 ± 135.6 ^{d}	2155.6 ± 106.1 ^{d}	2863.0 ± 483.0 ^{c}	5481.0 ± 386.0 ^{b}	11972.0 ± 243.0 ^{a}
Serine	78.0 ± 5.68 ^{d}	145.6 ± 65.6 ^{c}	361.6 ± 221.0 ^{a}	93.6 ± 2.26 ^{d}	264.8 ± 1.33 ^{b}	254.0 ± 1.00 ^{b}	49.0 ± 1.56 ^{e}	50.1 ± 1.84 ^{e}	53.7 ± 1.10 ^{e}
Valine	6.23 ± 0.19 ^{h}	10.1 ± 0.26 ^{ef}	11.5 ± 0.45 ^{d}	17.6 ± 0.25 ^{b}	24.7 ± 0.25 ^{a}	16.0 ± 0.14 ^{c}	7.93 ± 0.40 ^{g}	9.6 ± 0.42 ^{f}	10.6 ± 0.25 ^{e}

Threonine	6.01 ± 0.45 ^{f}	17.5 ± 0.24 ^{e}	27.8 ± 2.41 ^{d}	25.9 ± 0.44 ^{d}	79.4 ± 1.77 ^{a}	59.5 ± 0.33 ^{b}	25.2 ± 0.85 ^{h}	54.0 ± 1.17 ^{c}	55.4 ± 1.12 ^{c}

Metabolite	**04 DAH**			**10 DAH**			**21 DAH**		
	CTRL	***ETHY***	***MeJA***	***CTRL***	***ETHY***	***MeJA***	***CTRL***	***ETHY***	***MeJA***
Aspartic acid	1523.20 ± 54.20 ^{c}	1528.3 ± 48.0 ^{c}	2661.9 ± 58.7 ^{c}	2166.8 ± 58.7 ^{c}	60460.0 ± 776.0 ^{b}	119398.0 ± 2555.0 ^{a}	1540.8 ± 161.0 ^{c}	2817.0 ± 246.0 ^{c}	2649.3 ± 55.7 ^{c}
Glutamic acid	1744.3 ± 75.6 ^{b}	1848.4 ± 61.0 ^{b}	2952.7 ± 143.5 ^{b}	4906.7 ± 42.6 ^{b}	134180.0 ± 12596.0 ^{a}	130476.0 ± 628.0 ^{a}	3957.0 ± 424.0 ^{b}	7511.0 ± 224.0 ^{b}	6848.0 ± 148.1 ^{b}
Glutamine	185.1 ± 7.71 ^{f}	1837.0 ± 63.8 ^{d}	2928.8 ± 122.1 ^{c}	519.3 ± 9.24 ^{e}	10775.5 ± 100.4 ^{b}	15189.7 ± 38.1 ^{a}	475.8 ± 38.7 ^{e}	613.2 ± 10.4 ^{e}	290.9 ± 26.0 ^{f}
GABA	207.6 ± 17.2 ^{f}	3013.8 ± 75.1 ^{d}	5553.0 ± 415.0 ^{c}	1060.8 ± 45.9 ^{e}	14633.7 ± 97.0 ^{b}	15172.5 ± 79.6 ^{a}	216.9 ± 20.6 ^{f}	332.8 ± 8.86 ^{f}	301.4 ± 11.4 ^{f}
Asparagine	139. ± 6.22 ^{e}	152.9 ± 4.24 ^{e}	239.6 ± 11.5 ^{de}	260.2 ± 6.88 ^{d}	4599.6 ± 93.0 ^{a}	3160.6 ± 90.4 ^{b}	235.6 ± 11.7 ^{de}	440.2 ± 14.0 ^{c}	408.2 ± 10.9 ^{c}
Tryptophan	173. ± 7.10 ^{cd}	204.7 ± 6.39 ^{cd}	279.5 ± 11.4 ^{cd}	321.4 ± 8.65 ^{c}	5987.0 ± 328.0 ^{b}	9517.8 ± 65.3 ^{a}	27.1 ± 0.94 ^{d}	53.6 ± 3.28 ^{d}	58.9 ± 2.04 ^{cd}
Phenylalanine	7.64 ± 0.47 ^{f}	13.7 ± 0.18 ^{f}	18.8 ± 0.73 ^{f}	26.4 ± 1.32 ^{f}	606.8 ± 27.9 ^{d}	717.4 ± 4.43 ^{a}	50.9 ± 2.67 ^{e}	147.9 ± 4.86 ^{c}	77.6 ± 1.87 ^{d}
Tyrosine	21.1 ± 0.77 ^{e}	23.7 ± 0.90 ^{de}	26.2 ± 1.39 ^{de}	27.8 ± 2.37 ^{de}	109.1 ± 4.05 ^{a}	83.6 ± 3.37 ^{b}	62.3 ± 3.70 ^{c}	86.4 ± 6.21 ^{b}	28.9 ± 0.70 ^{d}
Total	4593.8 ± 170.8 ^{f}	9770.0 ± 289.0 ^{ef}	20268.0 ± 1025.0 ^{cd}	10593.8 ± 94.5 ^{def}	233987.0 ± 11964.0 ^{b}	296201.0 ± 2412.0 ^{a}	9512.0 ± 940.0 ^{ef}	17597.0 ± 548.0 ^{cde}	22755.0 ± 435.0 ^{c}
**D) Saturated fatty acids**									
Capric acid	61.9 ± 1.89 ^{c}	93.8 ± 4.32 ^{b}	133.4 ± 0.79 ^{a}	14.9 ± 0.41 ^{f}	28.9 ± 0.14 ^{e}	43.0 ± 1.41 ^{d}	6.77 ± 0.04 ^{g}	7.93 ± 0.26 ^{g}	10.0 ± 0.15 ^{g}
Lauric acid	40.5 ± 2.10 ^{c}	56.4 ± 2.24 ^{b}	83.1 ± 1.02 ^{a}	19.7 ± 1.16 ^{e}	30.3 ± 0.94 ^{d}	42.2 ± 1.44 ^{c}	6.82 ± 0.04 ^{g}	8.34 ± 8.27 ^{fg}	10.0 ± 0.17 ^{f}
Myristic acid	18.9 ± 0.83 ^{d}	42.1 ± 1.52 ^{b}	55.5 ± 1.60 ^{a}	15.0 ± 0.49 ^{e}	38.4 ± 0.30 ^{c}	56.1 ± 1.96 ^{a}	6.13 ± 0.07 ^{g}	8.73 ± 0.29 ^{f}	13.5 ± 0.24 ^{e}
Palmitic acid	436.7 ± 10.4 ^{cd}	470.6 ± 20.2 ^{b}	570.1 ± 16.2 ^{a}	413.1 ± 10.8 ^{d}	459.7 ± 7.05 ^{bc}	541.2 ± 22.8 ^{a}	159.6 ± 22.8 ^{f}	264.1 ± 13.1 ^{e}	451.2 ± 10.0 ^{bc}
Stearic acid	16.5 ± 0.49 ^{e}	20.1 ± 0.76 ^{d}	43.5 ± 10.9 ^{a}	14.6 ± 10.3 ^{f}	21.6 ± 0.18 ^{d}	31.6 ± 1.16 ^{b}	9.94 ± 0.18 ^{g}	14.7 ± 0.66 ^{ef}	27.2 ± 1.36 ^{c}
Eicosanoic acid	93.9 ± 1.59 ^{b}	90.6 ± 2.52 ^{b}	128.8 ± 8.70 ^{a}	10.2 ± 0.30 ^{de}	16.3 ± 0.35 ^{d}	24.7 ± 0.99 ^{c}	7.16 ± 0.17 ^{e}	11.0 ± 0.34 ^{de}	12.6 ± 0.34 ^{de}
Docosanoic acid	43.7 ± 1.04 ^{cd}	47.1 ± 2.02 ^{b}	57.0 ± 1.62 ^{a}	41.3 ± 1.09 ^{d}	45.9 ± 0.71 ^{bc}	54.1 ± 2.28 ^{a}	15.9 ± 0.15 ^{f}	26.4 ± 1.31 ^{e}	45.1 ± 1.00 ^{bc}
Tricosanoic acid	27.6 ± 1.15 ^{d}	30.3 ± 0.54 ^{c}	56.1 ± 2.65 ^{a}	20.3 ± 0.28 ^{f}	24.2 ± 0.22 ^{e}	36.4 ± 1.22 ^{b}	7.08 ± 0.17 ^{h}	13.8 ± 0.73 ^{g}	18.3 ± 0.43 ^{f}
Lignoceric acid	22.1 ± 1.82 ^{e}	32.6 ± 1.28 ^{c}	49.9 ± 1.05 ^{a}	13.9 ± 0.19 ^{g}	19.1 ± 0.50 ^{f}	38.5 ± 2.54 ^{b}	7.17 ± 0.24 ^{h}	13.3 ± 0.66 ^{g}	26.5 ± 0.67 ^{d}
Hyenic acid	32.5 ± 1.05 ^{c}	45.5 ± 1.67 ^{b}	53.9 ± 1.01 ^{a}	8.83 ± 0.21 ^{e}	17.2 ± 0.54 ^{d}	32.3 ± 1.32 ^{c}	6.58 ± 0.08 ^{f}	8.91 ± 0.32 ^{e}	10.0 ± 0.45 ^{e}
Cerotic acid	10.0 ± 0.71 ^{d}	15.9 ± 0.68 ^{c}	48.4 ± 1.06 ^{a}	10.2 ± 0.31 ^{d}	16.3 ± 0.35 ^{c}	24.7 ± 1.00 ^{b}	7.29 ± 0.16 ^{e}	8.75 ± 0.39 ^{de}	10.1 ± 0.18 ^{d}
Montanic acid	34.4 ± 1.33 ^{b}	35.0 ± 1.35 ^{b}	41.7 ± 0.93 ^{a}	9.49 ± 0.22 ^{f}	14.8 ± 0.15 ^{e}	23.9 ± 1.28 ^{c}	9.85 ± 0.16 ^{f}	16.9 ± 0.69 ^{d}	23.9 ± 0.44 ^{c}
Total	838.8 ± 20.6 ^{c}	979.8 ± 32.7 ^{b}	1321.4 ± 17.9 ^{a}	591.6 ± 14.5 ^{f}	732.6 ± 9.80 ^{d}	948.9 ± 38.3 ^{b}	250.4 ± 2.03 ^{h}	403.0 ± 18.7 ^{g}	658.7 ± 15.0 ^{e}
**E) Unsaturated fatty acids**									
Oleic acid	610.5 ± 26.0 ^{e}	1734.8 ± 99.8 ^{b}	2076.7 ± 43.4 ^{a}	339.4 ± 7.63 ^{g}	714.1 ± 22.9 ^{d}	1089.9 ± 45.7 ^{c}	213.0 ± 7.05 ^{h}	441.0 ± 26.0 ^{f}	581.2 ± 20.5 ^{e}
Linoleic acid	1079.5 ± 56.5 ^{d}	1570.6 ± 87.8 ^{c}	2704.4 ± 51.1 ^{a}	749.6 ± 21.4 ^{f}	922.6 ± 10.5 ^{e}	2173.2 ± 71.6 ^{b}	593.1 ± 5.06 ^{g}	706.4 ± 19.5 ^{f}	701.4 ± 20.3 ^{fg}
Linolenic acid	94.9 ± 4.36 ^{b}	99.9 ± 3.82 ^{b}	152.4 ± 2.46 ^{a}	24.0 ± 0.33 ^{f}	52.5 ± 0.89 ^{d}	70.4 ± 2.25 ^{c}	17.3 ± 2.32 ^{g}	31.9 ± 1.65 ^{e}	47.9 ± 1.04 ^{d}
Total	1784.9 ± 78.7 ^{c}	3405.3 ± 188.8 ^{b}	4933.6 ± 96.7 ^{a}	1113.1 ± 29.0 ^{e}	1689.3 ± 30.0 ^{c}	3333.5 ± 116.0 ^{b}	823.4 ± 11.1 ^{f}	1179.2 ± 46.0 ^{de}	1330.5 ± 41.2 ^{d}

Values were presented as normalized area by internal standards. CTRL: Control fruits. Different superscript letters indicate statistical significance ($p < 0.05$) at the same line (mean ± standard deviation. $n = 4$). GABA. γ-aminobutyric acid; DAH. days after harvest.
