# Supplementary material for: Induction of Metabolic Changes in Amino Acid, Fatty Acid, Tocopherol, and Phytosterol Profiles by Exogenous Methyl Jasmonate Application in Tomato Fruits
Source: Plants (Basel). 2022 Jan 28;11(3):366. doi: 10.3390/plants11030366 (PMC8838126; doi:10.3390/plants11030366)
Supplement: Supplementary file 1 [file plants-11-00366-s001.zip › plants-1525363-SI/Table S2_.pdf]

plants

Table S2. Carotenoids contents (µg g^{-1} FW) in tomato (*Solanum lycopersicum* L. cv. Grape) fruits exposed to ethylene (ETHY) and methyl jasmonate (MeJA) treatment at 04, 10 and 21 days after harvest (DAH) detected by high performance liquid chromatography (HPLC).

Metabolite	04 DAH			10 DAH			21 DAH		
	CTRL	*ETHY*	*MeJA*	*CTRL*	*ETHY*	*MeJA*	*CTRL*	*ETHY*	*MeJA*
Lycopene	15.4 ± 0.43 ^{i}	114.9 ± 1.86 ^{g}	56.5 ± 1.16 ^{h}	487.1 ± 4.10 ^{f}	684.5 ± 2.21 ^{d}	742.5 ± 0.63 ^{c}	653.6 ± 0.82 ^{e}	1115.3 ± 0.54 ^{a}	923.5 ± 0.99 ^{b}
β-carotene	3.87 ± 0.02 ^{h}	4.16 ± 0.03 ^{g}	4.26 ± 0.03 ^{f}	5.32 ± 0.10 ^{e}	6.23 ± 0.02 ^{d}	7.25 ± 0.03 ^{c}	7.23 ± 0.01 ^{c}	10.2 ± 0.03 ^{a}	9.13 ± 0.01 ^{b}
Lutein	2.43 ± 0.02 ^{i}	2.75 ± 0.01 ^{h}	3.09 ± 0.13 ^{g}	3.27 ± 0.06 ^{f}	3.66 ± 0.02 ^{e}	4.44 ± 0.03 ^{c}	3.93 ± 0.05 ^{d}	5.25 ± 0.03 ^{a}	5.03 ± 0.01 ^{b}
Total	21.7 ± 0.42 ^{i}	121.8 ± 2.18 ^{g}	63.8 ± 1.22 ^{h}	495.6 ± 4.10 ^{f}	694.4 ± 2.18 ^{d}	754.1 ± 0.63 ^{c}	664.7 ± 0.85 ^{e}	1130.7 ± 0.55 ^{a}	937.6 ± 1.00 ^{b}

CTRL: Control fruits. Different superscript letters indicate statistical significance ($p < 0.05$) at the same line (mean ± standard deviation, $n = 4$).
