# Supplementary material for: Induction of Metabolic Changes in Amino Acid, Fatty Acid, Tocopherol, and Phytosterol Profiles by Exogenous Methyl Jasmonate Application in Tomato Fruits
Source: Plants (Basel). 2022 Jan 28;11(3):366. doi: 10.3390/plants11030366 (PMC8838126; doi:10.3390/plants11030366)
Supplement: Supplementary file 1 [file plants-11-00366-s001.zip › plants-1525363-SI/Table S3_.pdf]

plants

Table S3. Tocopherols and phytosterols in tomato (*Solanum lycopersicum* L. cv. Grape) fruits exposed to ethylene (ETHY) and methyl jasmonate (MeJA) treatment at 04, 10 and 21 days after harvest (DAH) detected by gas chromatography-mass spectrometry (GC-MS).

Metabolite	04 DAH			10 DAH			21 DAH		
	CTRL	*ETHY*	*MeJA*	*CTRL*	*ETHY*	*MeJA*	*CTRL*	*ETHY*	*MeJA*
**A) Tocopherols**									
α-tocopherol	152.7 ± 14.5 ^{g}	337.4 ± 21.7 ^{ef}	313.3 ± 7.98 ^{f}	531.9 ± 12.1 ^{d}	704.5 ± 7.05 ^{b}	974.4 ± 43.8 ^{a}	375.8 ± 1.57 ^{e}	608.6 ± 25.5 ^{c}	701.2 ± 15.1 ^{b}
β-tocopherol	14.1 ± 0.27 ^{i}	22.8 ± 1.03 ^{g}	18.9 ± 0.41 ^{h}	62.3 ± 2.15 ^{c}	70.7 ± 0.56 ^{b}	91.6 ± 2.94 ^{a}	31.6 ± 0.23 ^{f}	47.2 ± 1.13 ^{e}	57.1 ± 1.36 ^{d}
γ-tocopherol	11.6 ± 0.49 ^{f}	65.3 ± 7.87 ^{c}	54.8 ± 0.87 ^{d}	14.6 ± 0.38 ^{f}	76.6 ± 4.67 ^{b}	91.1 ± 5.03 ^{a}	18.9 ± 0.72 ^{f}	33.9 ± 1.96 ^{e}	46.6 ± 1.09 ^{d}
Total	178.4 ± 15.1 ^{f}	425.5 ± 25.0 ^{e}	387.0 ± 9.19 ^{e}	608.9 ± 14.6 ^{d}	851.8 ± 8.11 ^{b}	1157.1 ± 45.3 ^{a}	426.4 ± 1.45 ^{e}	689.6 ± 26.5 ^{c}	804.9 ± 17.5 ^{b}
**C) Phytosterols**									
β-sitosterol	113.2 ± 7.14 ^{h}	244.8 ± 9.71 ^{f}	244.8 ± 4.04 ^{g}	410.2 ± 10.9 ^{d}	566.1 ± 4.20 ^{b}	750.6 ± 27.1 ^{a}	282.7 ± 1.32 ^{e}	466.7 ± 14.6 ^{c}	594.4 ± 14.1 ^{b}
Stigmasterol	227.3 ± 10.5 ^{f}	427.1 ± 10.7 ^{d}	326.6 ± 6.49 ^{e}	344.1 ± 8.75 ^{e}	528.2 ± 3.64 ^{b}	581.4 ± 21.5 ^{a}	400.8 ± 1.71 ^{d}	497.1 ± 15.6 ^{c}	554.8 ± 12.3 ^{ab}
Stigmastadienol	18.2 ± 0.26 ^{g}	31.2 ± 1.09 ^{e}	26.0 ± 0.50 ^{f}	27.8 ± 0.68 ^{f}	45.4 ± 0.72 ^{c}	55.1 ± 1.79 ^{a}	34.2 ± 0.16 ^{d}	43.7 ± 1.36 ^{c}	49.2 ± 1.18 ^{b}
Total	358.7 ± 14.3 ^{g}	703.1 ± 26.4 ^{e}	523.1 ± 10.9 ^{f}	782.0 ± 20.2 ^{d}	1139.7 ± 2.59 ^{b}	1387.1 ± 50.4 ^{a}	717.6 ± 2.80 ^{e}	1007.5 ± 31.5 ^{c}	1198.4 ± 27.6 ^{b}

Values were presented as normalized area by n-tridecane (internal non-polar standard). CTRL: Control fruits. Different superscript letters indicate statistical significance ($p < 0.05$) at the same line (mean ± standard deviation, $n = 4$).
